# Tracking the Asian tiger mosquito (*Aedes albopictus*) in Austria: benefits of combining expert-based monitoring with citizen science data

**DOI:** 10.1186/s13071-026-07574-z

**Published:** 2026-07-22

**Authors:** Karin Bakran-Lebl, Ian Kopacka, Barbara Seebacher, Heidi Bartel, Justin Catau, Barbara Eigner, Severin Falk, Johanna Gunczy, Gilbert Hafner, Karina Hauer-Lienhart, Peter Hufnagl, Hans Jerrentrup, Wolfgang Paill, Jana S. Petermann, Bernhard Rauchwarter, Julia Reichl, Thorsten Schwerte, Bita Shahi-Barogh, Maria Sophia Unterköfler, Katharina Vesely, Gernot Walder, Christian Wieser, Thomas Zechmeister, Klaus Zimmermann, Carina Zittra, David Zezula, Hans-Peter Fuehrer, Consortium Students of the Vetmeduni/mosquito group, Alena Bischof, Amelie Bittler, Kristin Demetz, Sebastian Paulic, Lea Perl, Laura Pürstl, Thomas Reinelt, Lisa Schlamadinger, Laura Schreier, Caroline Schultheiß, Robert Walsberger, Ute Wiesbauer, Laura Zehetbauer, Timo Zwettler

**Affiliations:** 1 Department for Vector-Borne Diseases, AGES - Austrian Agency for Health and Food Safety Ltd., 1090 Vienna, Austria; 2 Data, Statistics & Risk Assessment, AGES - Austrian Agency for Health and Food Safety Ltd., Zinzendorfgasse 27, 8010 Graz, Austria; 3 Department of Environment and Biodiversity, University of Salzburg, Hellbrunner Strasse 34, 5020 Salzburg, Austria; 4 Biological Station Lake Neusiedl, Seevorgelände 1, 7142 Illmitz, Austria; 5 Department for Biological Sciences and Pathobiology, Parasitology, University of Veterinary Medicine Vienna, Veterinärplatz 1, 1210 Vienna, Austria; 6 Center of Natural History, Zoology, Universalmuseum Joanneum, Weinzöttlstraße 16, 8045 Graz, Austria; 7 Verein Biologische Gelsenregulierung Entlang Thaya Und March, Rathausplatz 1, 2273 Hohenau an der March, Austria; 8 Department for Clinical Molecular Biology, AGES - Austrian Agency for Health and Food Safety Ltd., 1090 Vienna, Austria; 9 Institute of Zoology, Universiy of Innsbruck, Techniker Straße 25, 6020 Innsbruck, Austria; 10 Dr. Gernot Walder GmbH, Unterwalden 30, 9931 Außervillgraten, Austria; 11 Department for General Medicine, Johannes-Kepler-University Linz, Huemerstraße 3-5, 4020 Linz, Austria; 12 9064 Magdalensberg, Austria; 13 KTZ-Schädlingsprävention, Gilmstraße 18/1, 6850 Dornbirn, Austria; 14 Department of Functional and Evolutionary Ecology, University of Vienna, Djerassiplatz 1, 1030 Vienna, Austria

**Keywords:** *Aedes albopictus*, Austria, Citizen science, Invasive species, Monitoring, Mosquitoes, Ovitraps, Vectors

## Abstract

**Background:**

The Asian tiger mosquito (*Aedes albopictus*) is an invasive species and a competent vector of numerous pathogens. Its establishment in Austria represents a growing public health concern, underscoring the need for effective surveillance strategies. In Austria, two complementary monitoring systems are implemented, an expert-based ovitrap monitoring programme and a citizen science-based monitoring approach. This study investigates whether combining structured expert monitoring with citizen science reporting provides measurable benefits and identifies which advantages are most relevant.

**Methods:**

We analysed data from Austria’s nationwide ovitrap monitoring programme (following AIM-SURV protocol) and the citizen science project “Mosquito Alert” between 2020 and 2024. Both systems were evaluated at two spatial resolutions (5 × 5 km^2^ and 20 × 20 km^2^ grids) to quantify detection coverage, timing, and contribution to tracking spatial spread.

**Results:**

Combining ovitrap monitoring with the citizen science-based approach significantly improved detection coverage and spatiotemporal resolution. At fine scale (5 × 5 km^2^), the integrated approach increased positive grid cells by up to 70% compared to ovitraps alone, particularly in later years when citizen science reports dominated new detections. On the coarse scale (20 × 20 km^2^), ovitraps remained the primary source of early detections, while Mosquito Alert provided complementary signals in certain regions. Citizen science data were especially valuable for documenting local clustering, whereas ovitraps were more effective for identifying isolated new detections.

**Conclusions:**

Integrating structured expert surveillance with validated citizen science creates a more comprehensive and responsive monitoring framework for *Ae. albopictus*. This multi-source approach enhances early warning, spatial coverage, and operational decision-making, offering a scalable model for invasive vector surveillance in Europe.

**Graphical abstract:**

**Figure:**
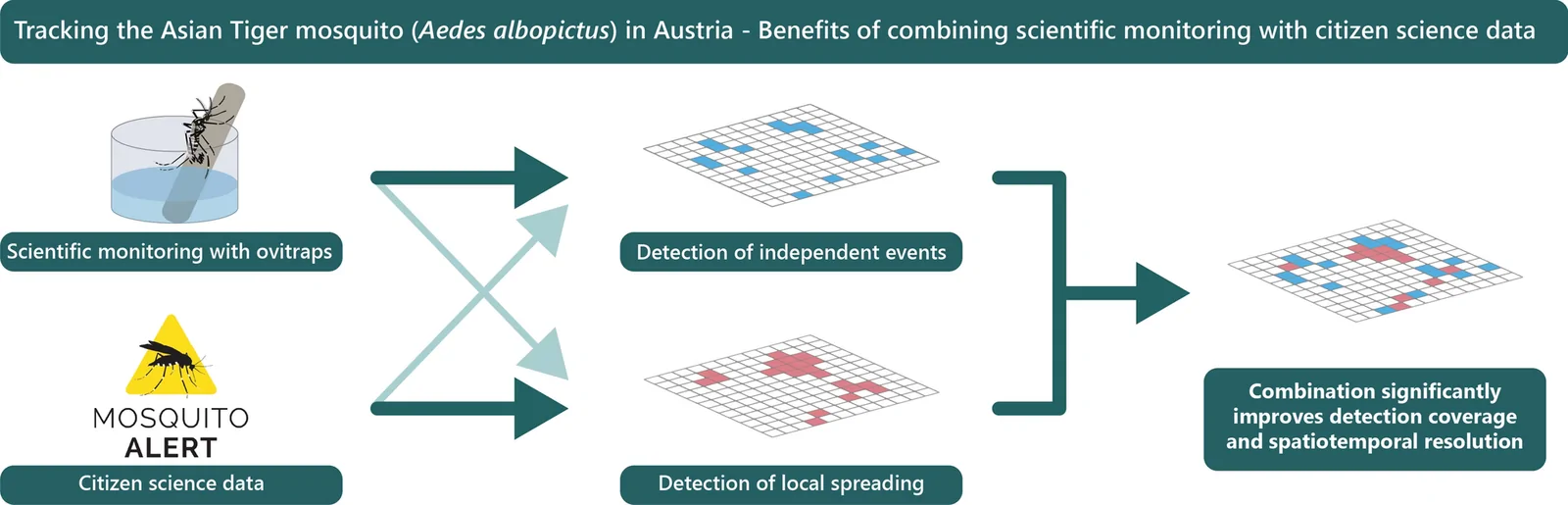

**Supplementary Information:**

The online version contains supplementary material available at https://doi.org/10.1186/s13071-026-07574-z.

## Background

Facilitated by global change, several exotic mosquito species, particularly container-breeding species of the *Aedes* genus, have been introduced to Europe in recent decades [1, 2]. Increased transport of goods and human mobility promote the unintentional introduction of these mosquitoes [3, 4]. Once in Europe, they can establish populations if suitable climatic conditions exist [2, 5]. Urbanization and climate warming are likely to enhance their breeding success and survival, expediting their establishment and spread [6–8]. Among these, the Asian tiger mosquito (*Aedes albopictus*) is of particular concern. Owing to its diurnal activity, it has a high contact rate with humans, rendering it not only a nuisance biter but also a significant public health threat as this species is a competent vector for a wide range of pathogens, including dengue virus, chikungunya virus, Zika virus, and *Dirofilaria* nematodes [1, 9–11]. Following its introduction, *Ae. albopictus* has been linked to several disease outbreaks with autochthonous transmissions in southern Europe [12, 13]. Particularly noteworthy is the recent considerable increase of dengue cases in Italy 2024 [14], France [15], and chikungunya cases in France 2025 [16].

In Austria, *Ae. albopictus* was first reported in 2012 [17]. In the subsequent years, this species was consistently found at various locations throughout the federal state Tyrol, particularly along the Inntal highway [18]. In 2020, it was documented in the capital city Vienna for the first time, followed by Graz in Styria [19] and Linz in Upper Austria [20] in 2021. Both are capital cities of federal states and, after Vienna, the next largest cities in Austria. To date, *Ae. albopictus* has been reported from all Austrian federal states, often at highway resting stations, and established populations are spreading in Vienna, Graz, and Linz.

The increasing establishment of *Ae. albopictus* in Austria heightens the risk of vector-borne diseases. To monitor the occurrence and spread of this species, a national monitoring programme was launched in 2020. The aim of this monitoring was the early detection of newly emerging populations and collected data on the spatial and temporal changes in the occurrence of *Ae. albopictus* and other non-native *Aedes* species, like *Ae. japonicus* and *Ae. koreicus*. While *Ae. japonicus* is already widespread in Austria, *Ae. koreicus* has so far only been reported sporadically [21].

The monitoring programme consists of two components. The first part is an expert-based monitoring programme, carried out using ovitraps and adhering to the AIM-SURV protocol [21, 22]. Ovitraps provide an ideal breeding habitat for container-breeding *Aedes* mosquitoes. These traps exploit the genus preference for laying eggs on moist substrates. Eggs are deposited on an oviposition support within the trap, which is collected and inspected for eggs at regular intervals [23]. Ovitraps offer a standardized, protocol-driven approach that ensures continuous data collection. Importantly, they not only provide information on species presence but also create absence records, which are essential for modelling spatial and temporal trends [22]. Their systematic placement at high-risk sites (e.g. urban hotspots, highway resting areas) enables early detection and quantification of egg densities, providing insights into population dynamics that opportunistic reporting cannot deliver. These attributes make ovitrap monitoring indispensable for establishing baselines and validating other data sources.

Although on average of 57 sites are sampled annually, there are large areas of the country where we do not have any information on the status of *Ae. albopictus*. To fill the gaps in expert monitoring, it is combined with the citizen science project Mosquito Alert [24]. Within this project, citizens can report invasive mosquito species such as *Ae. albopictus*, *Ae. koreicus*, *Ae. japonicus*, and *Ae. aegypti*—although the latter is, in Europe, currently known only from areas east of the Black Sea and from Cyprus—as well as important native vectors of pathogens from the *Culex pipiens* group, using a dedicated app. Mosquito Alert was originally launched in Spain in 2014, but was adapted to the European context in 2020 by expanding the species spectrum and making it available in 19 different languages. This enables the discovery of previously unknown populations and early detection of new species introductions. For example, Mosquito Alert helped detect the Asian bush mosquito, *Ae. japonicus*, in Spain for the first time [25]. Mosquito sightings are reported by uploading photos, which experts then evaluate to determine if the mosquito belongs to one of the target species and, if so, identify the specific species. Based on the visibility of identification features in the photo, a target species is classified as either "definite" or "probable". Reported mosquitoes are evaluated independently by three to four experts. Primarily, the evaluation is conducted by national experts of each participating country, which allows these experts to assess the significance of a finding and take action if necessary. Reported mosquitoes are displayed anonymously on a publicly accessible map, providing a valuable data source for health authorities and mosquito control programmes to identify areas requiring intervention. Citizen science participation has become an important tool for monitoring potential invasive mosquito species [26]. Those monitoring systems, like Mosquito Alert, expand spatial coverage beyond the fixed ovitrap network, leveraging citizen participation to detect occurrences in areas that would otherwise remain unsampled. Its expert validation process ensures high specificity, and the ability to capture first occurrences and local spread in real time adds significant operational value.

The aim of this study was to analyse and quantify the benefits of each monitoring system. Furthermore, we examined how the integration of these two programmes enhanced knowledge regarding the distribution and spread of *Ae. albopictus* in Austria. Because the confirmation of tiger mosquito presence within a specific area depends on the scale of the political unit considered, we additionally investigated how the outcomes of the two monitoring programmes varied with the spatial resolution of the political units applied.

## Methods

### Ovitrap monitoring

Since 2020, Austria has run a nationwide programme to monitor *Ae. albopictus* using ovitraps, following the AIM-SURV protocol [21, 22].

Monitoring sites were primarily selected in urban or suburban areas and locations where alien mosquitoes might be introduced into the country (e.g. airports, motorway service areas, train stations). Nationwide coverage and continuity are achieved by annually sampling 45–65 sites with ovitraps, with minor interannual variation in both site locations and the total number of collection sites (Table 1). At each site, traps were placed in five positions, though exceptions were made to cover specific areas (e.g. 21 traps at the airport). To prevent traps from influencing each other, they were placed 15–100 m apart and assigned to the same habitat. However, these standards were not always met due to limited availability of suitable trap locations. Trap positions were chosen to be shaded and moist (e.g. in bushes) to prevent drying out and provide suitable resting places for adult mosquitoes. The traps consist of a black 1.2-L plastic cup filled to three-quarters with tap water. As oviposition support, a wooden tongue depressor (15 × 1.8 cm) was used, which was attached to one side of the cup using a stainless-steel clamp.

**Table 1:** Yearly effort and results of the ovitrap monitoring and Mosquito Alert (data from Austria)

	2020	2021	2022	2023	2024
*Ovitrap monitoring*					
Number of sampling sites	45	50	67	57	65
Number of sites positive for *Ae. albopictus*	2	9	27	21	36
Number of sites positive for *Ae. japonicus*	32	39	57	53	60
*Mosquito Alert*					
Number of adult reports	15	257	1039	2936	4581
Number of definite *Ae. albopictus* reports	0	36	54	273	733
Number of probable *Ae. albopictus* reports	1	25	98	296	784
Number of definite *Ae. japonicus* reports	4	1	43	72	56
Number of probable *Ae. japonicus* reports	2	18	152	219	195

For Mosquito Alert, the number of adult reports include all reported mosquitoes, independent of species and identification certainty. Additionally, findings of *Ae. albopictus* and *Ae. japonicus* marked as “definitely” and “probably” are shown

From the beginning of May until the end of October the traps were sampled weekly, although deviations could occur, resulting in an average (± standard deviation) time interval of 7.8 ± 3.5 days between trap checks. At each sampling event, the water and the oviposition substrate were changed, and the inner surface of the container was wiped with a paper tissue.

### Mosquito Alert

Citizen reports of mosquito observations were obtained from the freely available Mosquito Alert database [24], obtained via the Mosquito Alert Data Portal (https://labs.mosquitoalert.com/metadata_public_portal). All of the records underwent verification by experts based on the photographs taken by the citizen scientists. For our analysis we used the reports where the species identification of the experts resulted in a “definite” identification of *Ae. albopictus* and *Ae. japonicus*. However, the latter species was used only for comparison (see Additional file 1). We included reports from Austria from the years 2020–2024.

### Data analysis

For each sampling event from each of the monitoring systems, the location and date were recorded. From the ovitrap monitoring we obtained from each sampling event whether *Ae. albopictus* was “present” or “absent”, from the Mosquito Alert we obtained present data only. To assess spatial patterns, we applied two official grid systems from Statistics Austria (“Regionalstatistische Rastereinheiten”; downloaded from https://data.statistik.gv.at/web/meta.jsp?dataset=OGDEXT_RASTER_1 on 3.11.2025): a 5 × 5 km^2^ grid, representing the median size of Austrian municipalities and a 20 × 20 km^2^ grid, approximating the size of Austrian districts. Using two grid sizes allowed us to evaluate how spatial resolution influenced detection outcomes. The finer grid provided a detailed view of local distribution patterns, while the coarser grid enabled the assessment of geographical distribution and monitoring effectiveness at a regional scale. Each ovitrap check and each citizen report was assigned to the corresponding grid cell. These analyses were also performed for *Ae. japonicus* (Additional file 1: Fig. S1, S2) to assess whether the observed patterns represent general effects or are specific to *Ae. albopictus*. Analyses were performed using the ovitrap monitoring system as the baseline. The relative benefit of supplementing it with Mosquito Alert data was calculated as

$${\mathrm{Relative}}\; {\mathrm{benefit}} = \frac{{{\mathrm{Number}}\;{\text{ of}} \;{\mathrm{grid}}\; {\mathrm{cells}}\; {\mathrm{with}}\;{\mathrm{MA}}\; {\mathrm{or}}\;{\text{ OT}} - {\mathrm{Number}}\;{\text{ of}}\;{\text{ grid}}\;{\text{ cells}}\;{\text{ with}}\; {\mathrm{OT}}}}{{{\mathrm{Number}}\; {\mathrm{of}}\; {\mathrm{grid}}\; {\mathrm{cells}}\; {\mathrm{with}}\; {\mathrm{OT}}}} \cdot 100,$$

where MA = Mosquito Alert and OT = ovitrap monitoring.

To compare timeliness, we calculated the time difference (in days) between detections by the two systems for grid cells where *Ae. albopictus* was reported by both during the observation period.

To assess spatial spread, we analysed the occurrence of new grid cells with detections per year. A grid cell was classified as new in a given year if it contained at least one detection in the respective monitoring system and had not shown detections in any previous year in either system. Multiple detections within the same grid cell and year were counted once per monitoring system. If there were detections for both systems within a given year, they were counted for both systems. New grid cells were further classified as isolated if they were not directly adjacent (including diagonal adjacency) to any grid cell with detections in either monitoring system in previous years; otherwise, they were considered adjacent. Under this definition, a grid cell monitored by ovitraps could be classified as adjacent based on prior detections from either ovitrap monitoring or Mosquito Alert.

To evaluate whether observed numbers of isolated new detections differed from expectations imposed by spatial geometry and monitoring opportunity, we conducted a simulation analysis. For each year, grid resolution, and monitoring system (ovitrap monitoring, Mosquito Alert, and the combined system), the observed number of new detections was randomly allocated to grid cells eligible for observation by the respective system in that year, excluding all grid cells with prior detections in either system. For ovitrap monitoring, eligible grid cells were defined as those containing at least one ovitrap location in the respective year. For Mosquito Alert, eligible grid cells were restricted to cells with at least one mosquito notification in the respective year, regardless of species, thereby accounting for spatial variation in reporting effort. For the combined system, eligible grid cells comprised the union of grid cells eligible for either monitoring system. New detections were classified as isolated using the same adjacency definition as applied to the observed data. This procedure was repeated 10,000 times per scenario to generate null distributions of the expected number of isolated new detections under random placement. Observed values were compared with the simulated distributions using the median and the 2.5% and 97.5% quantiles.

Depending on year, between 53 and 544 grid cells were eligible for new detections across the combined surveillance system on the 5 × 5 km grid, of which 0–19.3% had at least one neighbouring cell with prior detections (with 0% occurring by definition in the first year). Corresponding values on the 20 × 20 km grid ranged from 34 to 139 eligible grid cells, with 0–47.4% having at least one neighbouring cell with prior detections. For ovitrap monitoring alone, the number of grid cells eligible for new detections was substantially smaller at both spatial resolutions, reflecting the fixed and spatially sparse layout of the ovitrap network; depending on year, 29–50 grid cells (5 × 5 km) and 17–33 grid cells (20 × 20 km) were eligible, of which 0–32.3% and 0–76.5%, respectively, had at least one neighbouring cell with prior detections.

All analyses were conducted in R (version 4.3.3) [27].

## Results

### Benefit of combining the monitoring systems

**Fig. 1:**
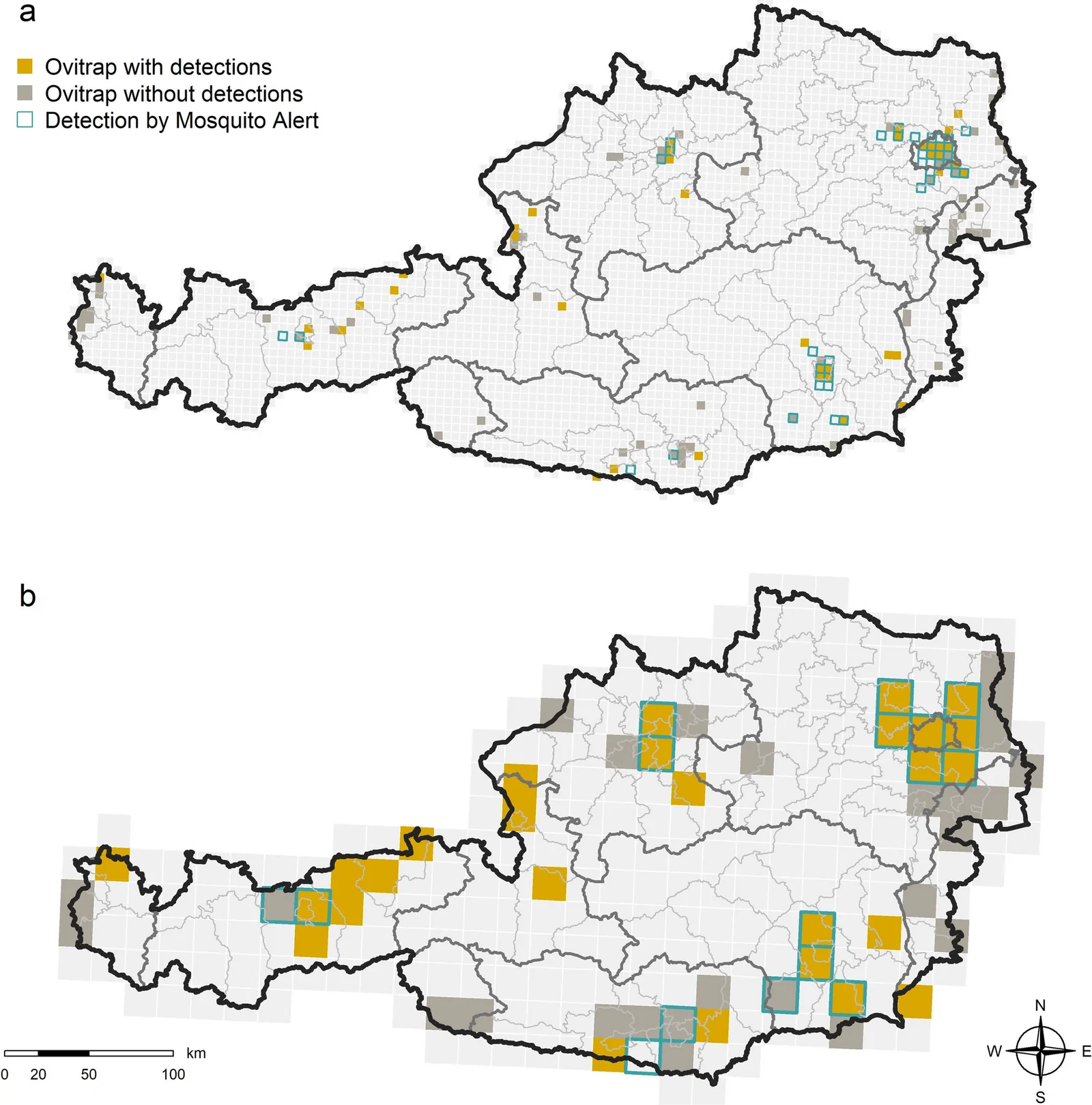
Number of cells with findings of *Ae. albopictus* in Austria during 2020–2024. Panel **a** showing the small-scale grid (5 × 5 km^2^) and panel **b** the large-scale grid (20 × 20 km^2^)

During 2020–2024, using the small-scale grid of 5 × 5 km^2^, *Ae. albopictus* could be detected with the ovitrap monitoring in 40 grid cells. As traps were deployed in 103 of these cells, this corresponds to a detection rate of 40.8%. During the same period, 42 positive cells were recorded with the Mosquito Alert. Although those numbers were similar, they did only partly overlap, resulting in a combined coverage of 67 grid cells (Fig. 1a).

**Fig. 2:**
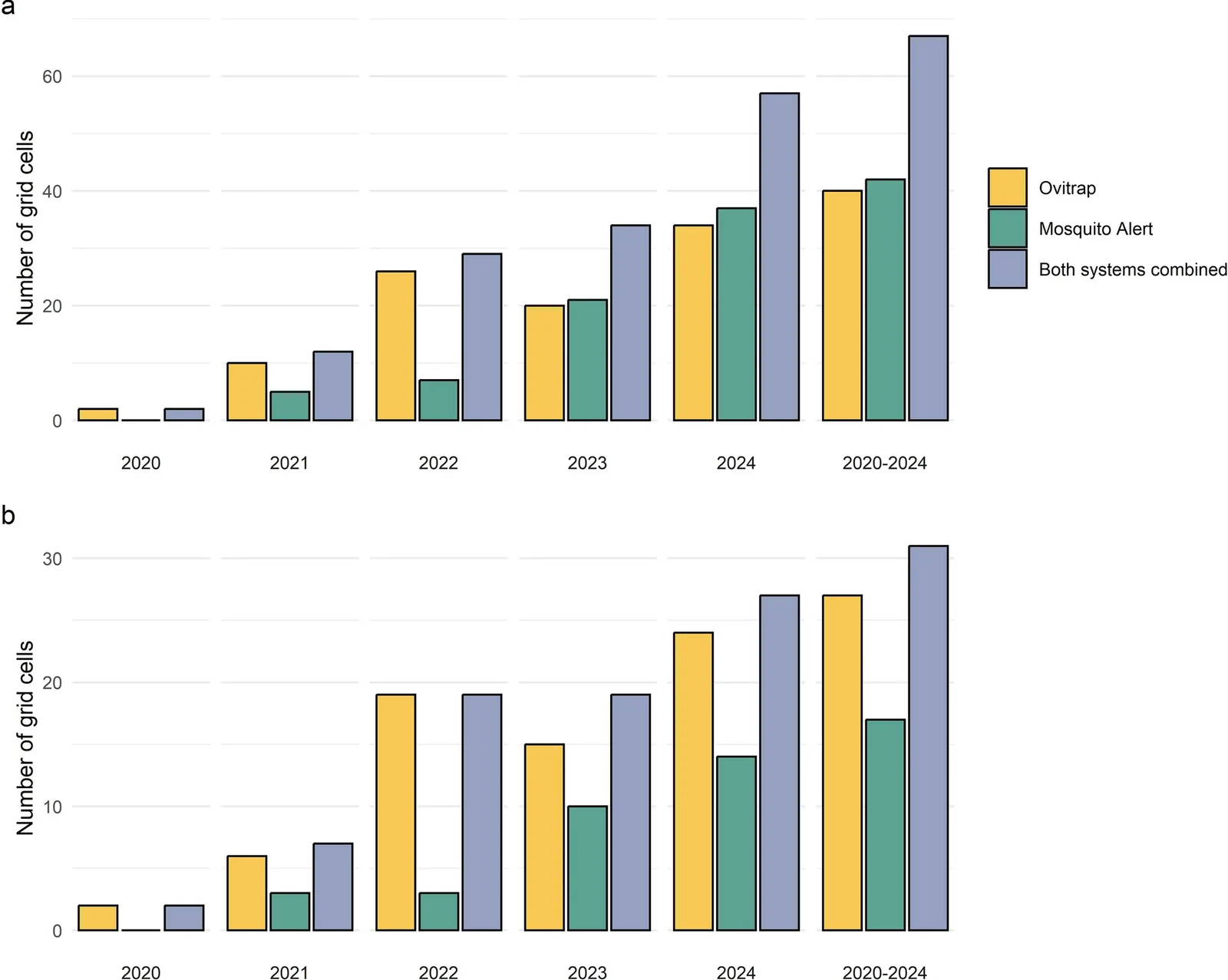
Number of grid cells at **a** small scale—5 × 5 km^2^ and **b** large scale—20 × 20 km^2^, with reported mosquito findings for ovitraps and Mosquito Alert over the years 2020—2024; the bars on the right represent cumulative reports for the entire period

Throughout the observation period, the number of cells with findings of *Ae. albopictus* increased in the small-scale grid from 2 in 2020 to 57 in 2024. During the initial period (2020–2022), most detections were attributable to ovitrap monitoring, whereas in 2023–2024, the majority of positive grid cells resulted from the citizen science project (Fig. 2a). Except for 2020, where no *Ae. albopictus* were reported with Mosquito Alert, the combination of the system consistently increased the spatial coverage of detections compared to ovitrap monitoring alone (Table 2). Over the study period, the combined approach identified 67 grid cells with detections, compared to 40 grid cells detected by ovitraps alone, corresponding to a relative benefit of 67.5%.

**Table 2:** Number of grid cells with detections of *Ae. albopictus* at small (5 × 5 km^2^) and large (20 × 20 km^2^) spatial scales, reported by the two monitoring systems separately and combined per year and cumulatively (2020–2024)

	2020	2021	2022	2023	2024	2020–2024
*Small scale*						
Ovitraps	2	10	26	20	34	40
Mosquito Alert	0	5	7	21	37	42
Combined	2	12	29	34	57	67
Benefit (%)	0.0	20.0	11.5	70.0	67.6	67.5
*Large scale*						
Ovitraps	2	6	19	15	24	27
Mosquito Alert	0	3	3	10	14	17
Combined	2	7	19	19	27	31
Benefit (%)	0.0	16.7	0.0	26.7	12.5	14.8

Furthermore, the relative benefit for combining both systems in comparison to using only the ovitrap monitoring is depicted

With the large-scale grid (20 × 20 km^2^), where ovitraps were deployed in 53 grid cells, tiger mosquitoes could be detected in 27 (detection rate of 50.1%), while only 17 were positive with the citizen science project. Combining the two monitoring programmes resulted in 31 positive large-scale grid cells (Fig. 1b). The number of cells positive for tiger mosquitoes increased from 2 in 2020 to 27 in 2024. At this larger scale, the number of positive cells generated by the ovitrap monitoring exceeded those from the Mosquito Alert project in all years (Fig. 2b). The relative benefit of adding the Mosquito Alert results to the ovitrap monitoring was less pronounced on this scale (Table 2), and over the study period an aggregated relative benefit of 14.8% could be achieved in comparison to using the ovitrap monitoring alone.

In comparison, *Ae. japonicus* was more widespread (Fig. S1). This species was already detected at high numbers in ovitraps during the early years of the monitoring programme, and its reporting frequency by Mosquito Alert has remained relatively stable since 2022. Despite these differences, the combined use of both monitoring systems provides a similarly benefit for this species as well (Fig. S2).

### Time difference in the detection of *Ae. albopictus*

**Fig. 3:**
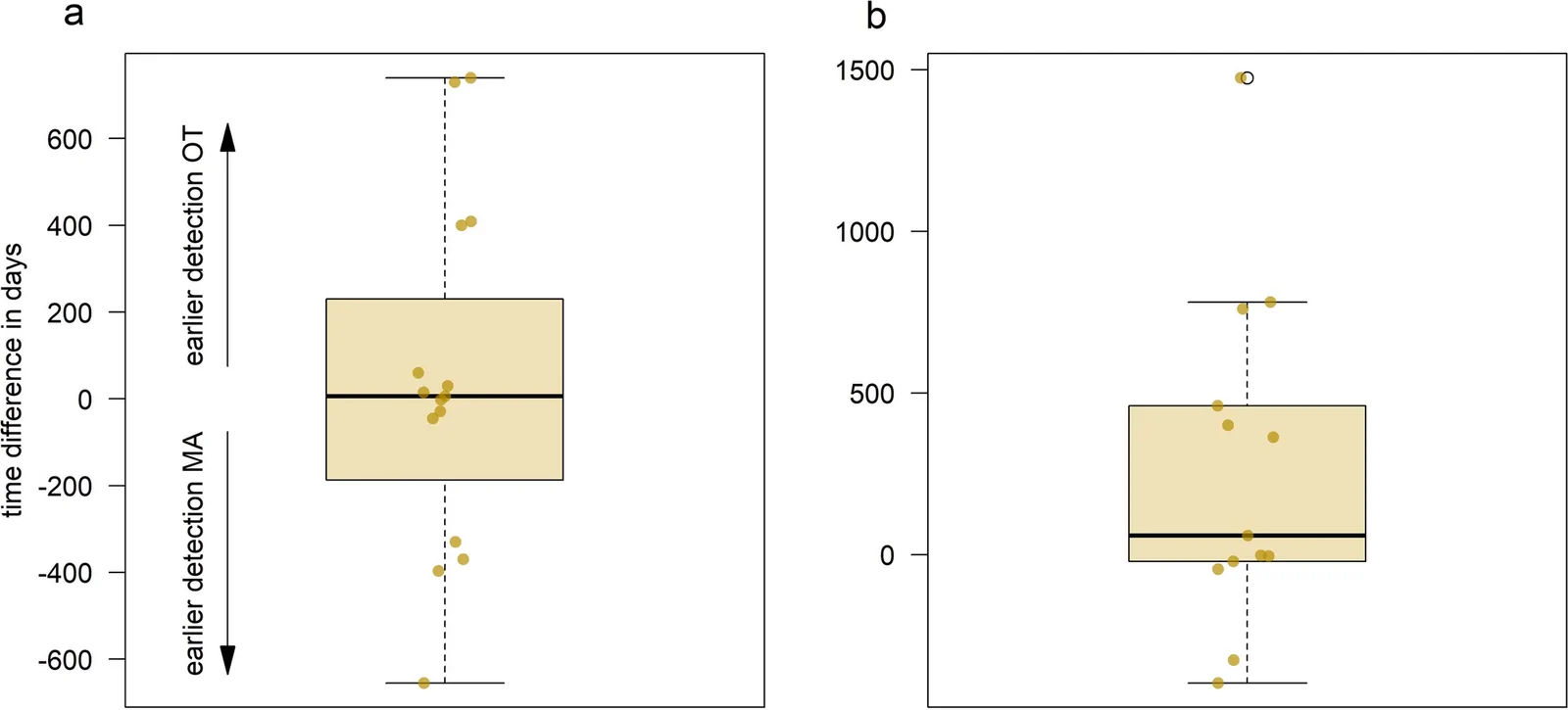
Time lag (in days) between the first detection by ovitraps and by Mosquito Alert within a given grid cell on small scale (**a**) and large scale (**b**). Only grid cells in which *Ae. albopictus* was detected by both monitoring systems during the observation period were included in the analysis. The difference was calculated as the date of the detection by Mosquito Alert minus the date of detection by ovitrap. Thus, positive values indicate earlier detection by ovitraps, while negative values indicate earlier detection by Mosquito Alert. Boxplots show the median and interquartile range, with whiskers extending to the most extreme values within 1.5 × IQR; individual data points are overlaid and jittered horizontally for visibility

The distribution of time differences between the first detections by ovitraps and Mosquito Alert reveals an overall balanced pattern at the fine-scale grid level. For grid cells where *Ae. albopictus* was detected by both monitoring systems, the interquartile range spans from 60 days earlier detection by ovitraps to 45 days earlier detection by Mosquito Alert (Fig. 3.a). This indicates that, in most cases, reports from both systems occur in close temporal proximity. Nevertheless, the time difference can occasionally be much larger, ranging from 740 days earlier by ovitraps to 650 days earlier by Mosquito Alert. Overall, these findings indicate that, at this smaller spatial resolution, both systems respond to the occurrence of *Ae. albopictus* at approximately the same speed, although local variations may occur.

The distribution of time differences between the first detections by ovitraps and Mosquito Alert at the 20 km grid level shows a pronounced asymmetry in favour of the ovitrap monitoring (Fig. 3b). In most grid cells where both systems reported detections, the first observation was made by ovitraps, sometimes with substantial lead times of several hundred days (up to 1475 days). Nevertheless, the distribution also includes individual grid cells where Mosquito Alert reported earlier detections (up to 397 days). This highlights that the citizen science monitoring provides complementary information even at the 20 km scale by reacting earlier in certain regions.

### Monitoring of the spatial spread

**Fig. 4:**
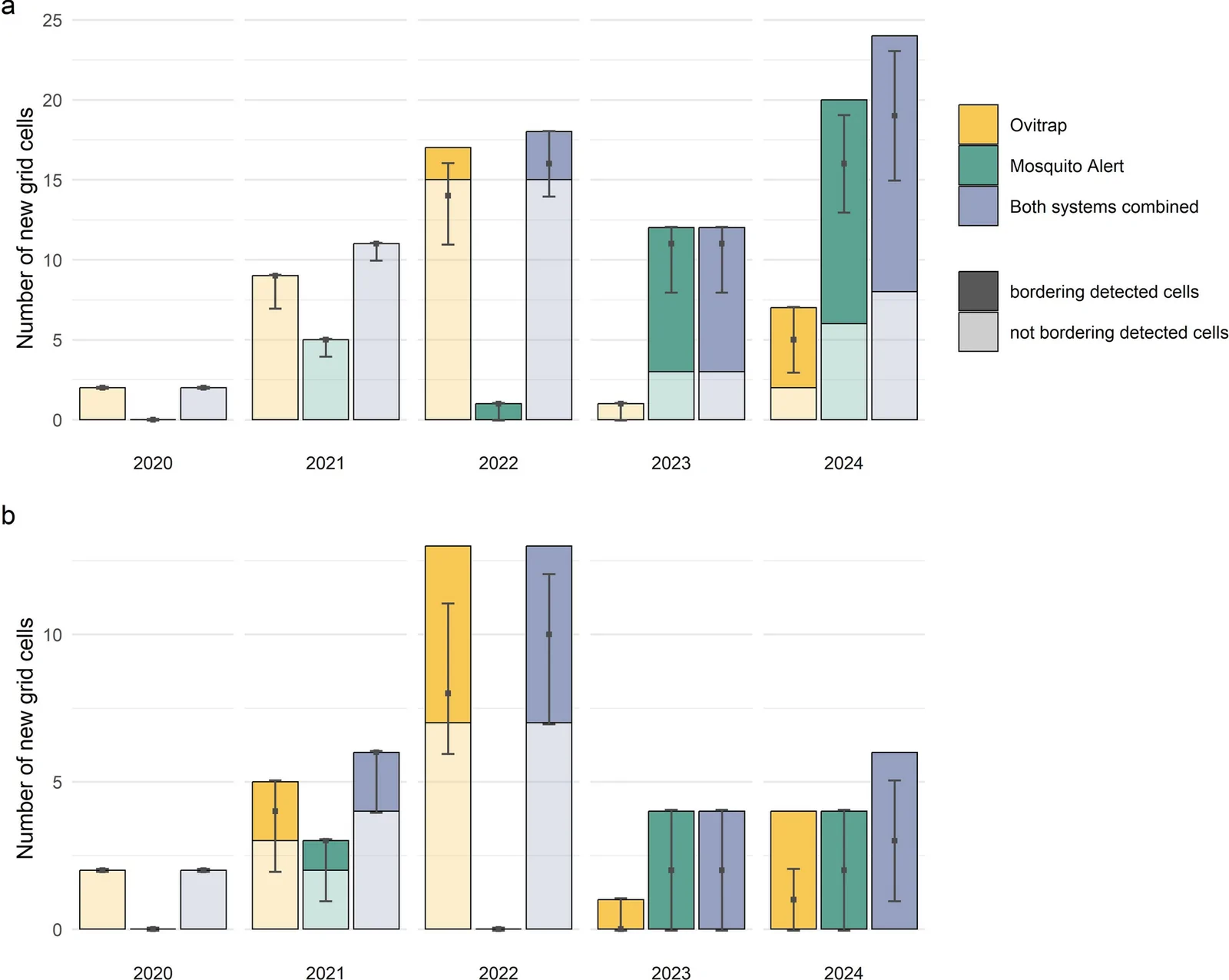
Annual dynamics of the spatial expansion of *Aedes albopictus* based on newly occupied grid cells on a small scale (**a**) and large scale (**b**). For each year, the number of grid cells with first reports—i.e. cells without previous detections—was determined. New cells adjacent to previously reported cells, indicating likely local spread, are marked without transparency. New cells occurring in isolation, without neighbouring detections in prior years, which may suggest independent introduction events or previously undetected populations are indicated by transparent colours. Small squares give the median of the expected (simulated) number of new isolated new grid cells; the error bars represent the interval within which 95% of the simulation results fall (2.5th–97.5th percentiles)

The comparative analysis of both systems’ capacity to detect new areas with occurrences of *Ae. albopictus* indicates temporal variability. Within the small-scale grid, initial detections between 2020 and 2022 were predominantly attributable to ovitrap monitoring. The peak performance of this method occurred in 2022, when 17 new grid cells were identified, compared to a single detection by Mosquito Alert. This pattern reversed in subsequent years: in 2023 and 2024, Mosquito Alert became the primary contributor to the identification of new areas, detecting 12 and 20 new grid cells, respectively, whereas ovitrap monitoring accounted for only one and seven detections in those years (Fig. 4a).

On the small‑scale grid, all new detections in 2020 and 2021, as well as most detections in 2022, occurred in grid cells that were not adjacent to previously reported occurrences (Fig. 4a). Simulation results indicate that, during these early years, the observed number of isolated new detections was within the range expected under random placement given the limited number of previously positive cells. In contrast, in 2023 and 2024 the number of isolated new detections was markedly lower than expected under random placement when considering both monitoring systems combined (Fig. 4a). Specifically, only 25% of new detections in 2023 and 33.3% in 2024 occurred in isolated grid cells, whereas simulations suggested substantially higher numbers of isolated detections would be expected by chance alone. The remaining detections (75% in 2023 and 66.6% in 2024) were found in grid cells adjacent to previously reported *Ae. albopictus* occurrences, indicating non‑random spatial clustering.

Notably, on this small scale, information on isolated new occurrences was mainly (67.4%) given by ovitraps, while information on spatial clustering was predominantly obtained through the Mosquito Alert project (77.4%). Simulation results show that, particularly for Mosquito Alert in 2023 and 2024, the observed number of isolated new detections was substantially lower than expected under random placement, indicating that the spatial pattern of reports was strongly influenced by proximity to previously detected occurrences. On the large‑scale grid, the pattern of new reports was similar to that observed on the small‑scale grid during the years 2020–2022 (Fig. 4b). Simulation results indicate that the number of isolated new detections during this period was largely consistent with expectations under random placement. In contrast, in 2023 and 2024, all newly reported *Ae. albopictus* occurrences were located in grid cells adjacent to cells with previous detections, and the observed number of isolated new detections was lower than expected under random placement for all monitoring systems (Fig. 4b). At this spatial resolution, new reports of *Ae. albopictus* occurrences were mainly attributable to ovitrap monitoring (85.7%), while the contribution of Mosquito Alert was considerably lower than on the small‑scale grid, particularly in 2023 and 2024.

## Discussion

Our analysis demonstrates that integrating structured expert surveillance (ovitraps) with validated citizen science (Mosquito Alert) yields a more complete, more responsive, and operationally useful picture of *Ae. albopictus* distribution and spread in Austria. Across 2020–2024, the two systems were complementary rather than redundant. Importantly, this period spans the transition from sporadic, isolated detections of *Ae. albopictus* to the establishment of stable populations in two major cities. The number of ovitraps was comparable during the study period. Findings from those traps dominated early detections during the initial phase of spread, a time when the presence of the Asian tiger mosquito, its associated risks, and the option to report sightings were largely unknown to the public. The number of citizen science reports increased substantially—approximately exponentially—over the study period [28]. This was driven by a combination of increased public awareness of the tiger mosquito problematic, a rising tiger mosquito burden, and intensified app promotion and media coverage [29, 30]. Due to the increasing number of reports, particularly from already affected areas, the relevance of the citizen science project has grown substantially over the years, and Mosquito Alert became increasingly important for identifying newly affected areas and documenting local expansion. These patterns held differently across spatial scales (5 × 5 km^2^ vs 20 × 20 km^2^ grids), highlighting that surveillance performance depends on both the method and the spatial resolution applied.

However, the ovitrap monitoring as well as the citizen science approach have limitations. Although ovitrap monitoring is rather cheap regarding the material costs, it is resource intensive as it requires regular inspections by trap operators. It is further geographically constrained, as this monitoring leaves large areas unsampled. Mosquito Alert, while broad in reach, depends on uneven citizen participation, which can introduce spatial and socio-demographic biases [31]. Additionally, the validation of citizen reports by multiple experts represents a considerable workload, especially as the number of submissions continues to increase, thus posing challenges for scalability. Future integration of AI-based image recognition and automated classification could help alleviate this burden and accelerate response times [32]. Furthermore, our analysis conservatively considered only reports in the category “definitely” (as identified by experts) from Mosquito Alert, which represented 47.7% of the *Ae. albopictus* reports in Mosquito Alert, prioritizing precision over sensitivity. Future work could quantify the trade-off gained by incorporating reports classified as “probably”.

This benefit of the combined monitoring was not limited to *Ae. albopictus* but was also evident for *Ae. japonicus*. This species was already widespread in Austria at the beginning of the monitoring programme and was consistently detected in ovitraps throughout the observation period [19]. Although *Ae. japonicus* is by far the most common species in the ovitrap monitoring, Mosquito Alert reports were dominated by *Ae. albopictus*. Compared to *Ae. japonicus*, *Ae. albopictus* is generally considered to cause a higher biting nuisance due to its stronger association with humans and urban environments [33], which likely explains the higher number of Mosquito Alert reports for *Ae. albopictus*.

Since 2022, when the Mosquito Alert app became better known to the public, the reports from this species have remained relatively stable over the following years. Despite its earlier establishment in Austria and its broader, less urban-focused distribution, the combined monitoring approach also provided a clear advantage for the monitoring of this species. This demonstrates that the observed benefit of the combined monitoring approach is a general effect across species.

Our analysis shows that the contribution of ovitrap monitoring and citizen science differs markedly depending on whether the goal is to detect independent introductions or track local expansion. Using adjacency (one-cell distance) as a proxy, early years (2020–2022) were dominated by non-adjacent detections. Simulation results indicate that, during this period, the observed number of isolated detections was largely consistent with expectations under random placement, given the limited number of previously positive grid cells. These early detections were primarily captured by ovitraps, reflecting their strategic placement at high-risk entry points such as motorway resting stations. The small number of findings in new grid cells by Mosquito Alert is likely caused by low *Ae. albopictus* occurrence in combination with the low participation in the citizen science project in those early years. In contrast, later years (2023–2024) were characterized by a predominance of adjacent detections. Simulation analyses showed that the number of isolated new detections during these years was substantially lower than expected under random placement, indicating spatial clustering beyond that imposed by grid geometry and monitoring opportunity. During this phase Mosquito Alert played an increasingly important role, especially at fine spatial resolution (5 × 5 km^2^). This shift suggests a transition from entry screening to monitoring the expansion boundary, where citizen science signals become particularly informative, reflecting its responsiveness in densely populated regions. The limited ability of ovitraps to detect fine-scale local spread is largely a consequence of the surveillance design itself: traps are positioned predominantly in high-risk areas and at potential points of entry, in accordance with the AIMSurv protocol [22]. It can be expected, that deploying additional ovitraps in a systematic grid pattern would enhance their capacity to capture local expansion dynamics. While such an approach could be valuable in small, localized studies aimed at generating more consistent and comparable data than citizen science reports, it would be impractical for nationwide surveillance due to the substantial logistical effort and costs involved.

When *Ae. albopictus* was detected by both ovitrap monitoring and Mosquito Alert within the same grid cell, the timing of first detection varied considerably and depended on spatial resolution. For cells detected by both systems, ovitraps were often earlier at 20 km scale (sometimes by > 1 year), while Mosquito Alert occasionally led by several months. This suggests that at this spatial resolution ovitraps play a stronger role in early detection. At 5 km scale, detections were closer in time, with both systems capable of early signals for different reasons (targeted risk sites vs high app usage). This again underscores the advantage of combining both systems, as their complementary strengths reduce the likelihood of missing early colonization events. Because the number of *Ae. albopictus* reports submitted via Mosquito Alert reflects a combination of true occurrence and changing levels of public participation; the contribution of the citizen science system to early detection is expected to vary over time. Other studies confirm that data provided by citizen scientists on the—often first—occurrence of potentially invasive mosquito species are of great value because they substantially enhance spatial coverage, enable early detection of introductions, and provide complementary information that would be difficult to obtain through expert monitoring alone [19, 25, 34–37]. However, the participation levels necessary to achieve such important findings need to be taken into account. While the number of ovitraps is expected to remain relatively stable in the coming years, public awareness of tiger mosquitoes and of the Mosquito Alert app will likely continue to grow. Consequently, Mosquito Alert may play an increasingly important role in detecting new occurrences in the future.

A limitation of this study is the reliance on adjacency at one-cell distance as a proxy for local spread vs independent introduction. While adjacency provides a practical operational proxy, it cannot fully disentangle secondary spread from repeated introductions. However, the use of simulation‑based null models allows the observed spatial patterns to be interpreted in light of the underlying monitoring geometry and effort. Fine-scale genetic approaches based on close-kin relationships have proven particularly useful for distinguishing local spread from repeated introductions, as they allow inference of dispersal and relatedness at very small spatial scales [38–40].

Our findings highlight that spatial resolution strongly shapes the apparent performance of monitoring systems. For local public health authorities, it is essential to be aware of both monitoring systems and their respective strengths and limitations associated with different spatial scales (e.g. district versus municipality) and that relying on a single system may provide an incomplete picture of *Ae. albopictus* occurrence within a given administrative unit. At fine resolution (5 × 5 km^2^ grids, in Austria approximately the size of a municipality), both ovitrap monitoring and citizen science reports contributed substantially to detecting spatial clustering, while Mosquito Alert became particularly effective in later years for identifying detections in grid cells adjacent to previously known occurrences, reflecting the ability of opportunistic observations to capture micro-scale expansion that structured networks may miss. During those later years of the study period, reporting activity increases, and newly occupied cells are more likely to be adjacent simply due to the growing number of detections. Thus, part of the observed pattern may arise from this temporal increase in participation rather than from biological spread alone. However, our simulation-based results confirm that these patterns deviate from random expectations and are not solely driven by grid structure or reporting opportunity, suggesting that the enhanced performance of the combined monitoring system reflects a genuine complementary effect rather than an artefact of participation growth. Conversely, at coarse resolution (20 × 20 km^2^, in Austria approximately the size of a district), ovitrap monitoring dominated detections, especially early introductions, because traps are strategically placed at high-risk entry points. Aggregation at larger scales tends to mask the spatiotemporal detail provided by citizen science, reducing its apparent contribution to early warning. Furthermore, looking at the time difference in the detection, at small scale, ovitrap and citizen science detections were temporally balanced, while aggregating data to coarser grids amplifies again the signal from ovitrap monitoring, which often appears substantially earlier than citizen science reports. These scale effects caution against direct comparisons across resolutions and underscore the need for multi-scale approaches in monitoring design [41].

## Conclusions

In conclusion, combining ovitrap monitoring with expert-validated citizen science materially improves the detection of newly colonized areas and the characterization of spatial spread, provided that analyses are mindful of scale, participation bias, and method-specific strengths. As European guidance moves towards harmonized, open data surveillance systems, Austria’s integrated approach offers a practical template for early warning and for targeted control of *Ae. albopictus*.

## Supplementary Information

Additional file 1 (PDF 360 KB)

## Data Availability

Data from the Mosquito Alert project are available from the Mosquito Alert Data Portal: https://labs.mosquitoalert.com/metadata_public_portal/meta_ipynb/reports.html. The ovitrap datasets are available on GBIF [42–46].
