# Supplementary material for: Tracking the Asian tiger mosquito (Aedes albopictus) in Austria: benefits of combining expert-based monitoring with citizen science data
Source: Parasit Vectors. 2026 Jul 22;19:390. doi: 10.1186/s13071-026-07574-z (PMC13563954; doi:10.1186/s13071-026-07574-z)
Supplement: Supplementary file 1 [file 13071_2026_7574_MOESM1_ESM.pdf]

Supplement

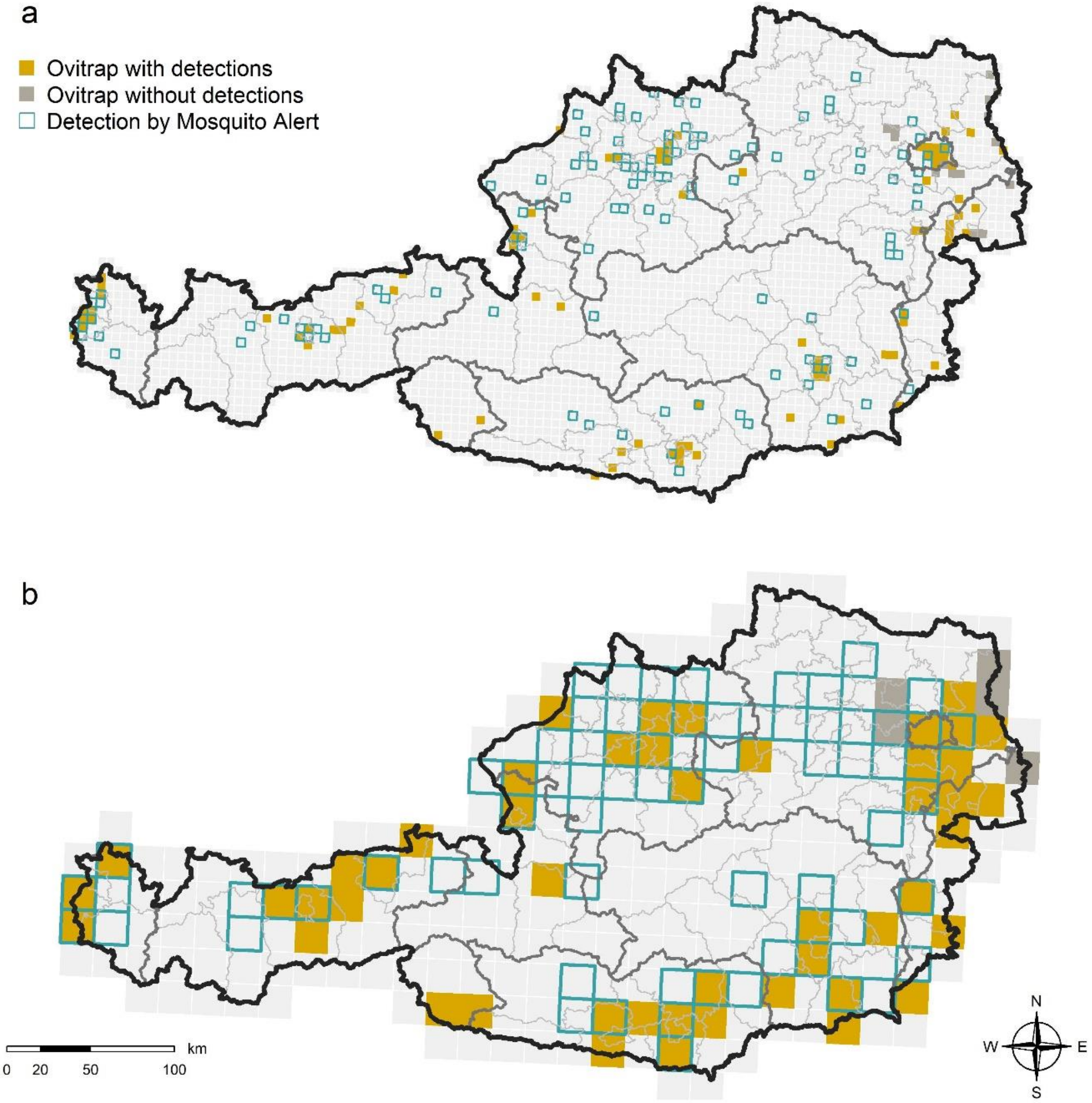

Figure S1. Number of cells with findings of *Aedes japonicus* in Austria during 2020-2024. Panel (a) showing the small-scale grid (5 × 5 km^2) and panel (b) the large-scale grid (20 × 20 km^2).

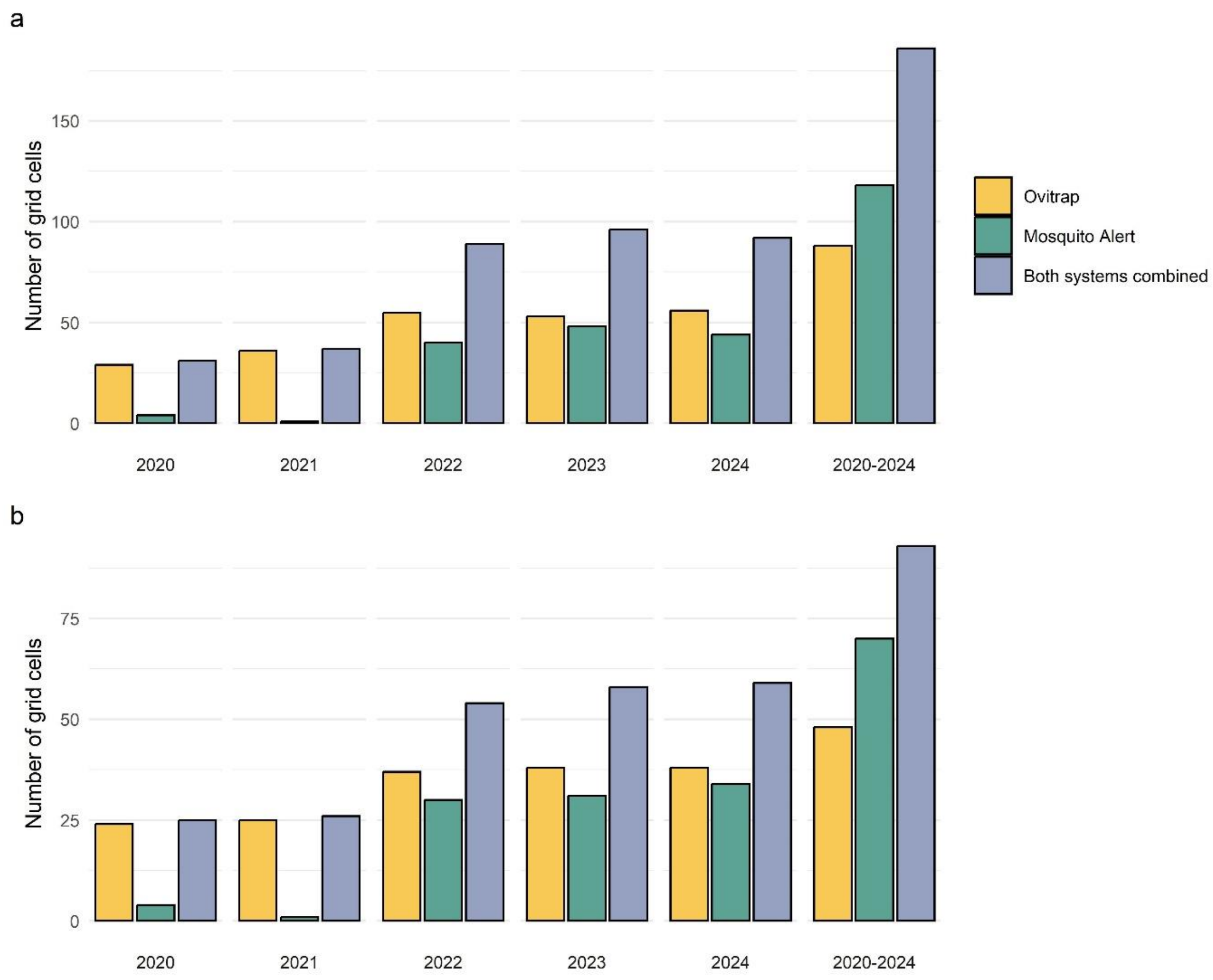

Figure S2. Number of grid cells at (a) small scale - 5 × 5 km^2 and (b) large scale - 20 × 20 km^2, with reported mosquito findings of *Aedes japonicus* for ovitraps and Mosquito Alert over the years 2020 - 2024; the bars on the right represent cumulative reports for the entire period.
